# A Perfusion MRI Study of Emotional Valence and Arousal in Parkinson's Disease

**DOI:** 10.4061/2011/742907

**Published:** 2011-09-28

**Authors:** Sunsern Limsoontarakul, Meghan C. Campbell, Kevin J. Black

**Affiliations:** ^1^Department of Psychiatry, Washington University School of Medicine St. Louis, St. Louis, MO 63110, USA; ^2^Department of Neurology, University of British Columbia, Vancouver, BC, Canada V6T 2B5; ^3^Department of Neurology, Washington University School of Medicine St. Louis, St. Louis, MO 63110, USA; ^4^Department of Radiology, Washington University School of Medicine St. Louis, St. Louis, MO 63110, USA; ^5^Department of Anatomy & Neurobiology, Washington University School of Medicine St. Louis, St. Louis, MO 63110, USA

## Abstract

*Background*. Brain regions subserving emotion have mostly been studied using functional magnetic resonance imaging (fMRI) during emotion provocation procedures in healthy participants. *Objective*. To identify neuroanatomical regions associated with spontaneous changes in emotional state over time. *Methods*. Self-rated emotional valence and arousal scores, and regional cerebral blood flow (rCBF) measured by perfusion MRI, were measured 4 or 8 times spanning at least 2 weeks in each of 21 subjects with Parkinson's disease (PD). A random-effects SPM analysis, corrected for multiple comparisons, identified significant clusters of contiguous voxels in which rCBF varied with valence or arousal. *Results*. Emotional valence correlated positively with rCBF in several brain regions, including medial globus pallidus, orbital prefrontal cortex (PFC), and white matter near putamen, thalamus, insula, and medial PFC. Valence correlated negatively with rCBF in striatum, subgenual cingulate cortex, ventrolateral PFC, and precuneus—posterior cingulate cortex (PCC). Arousal correlated positively with rCBF in clusters including claustrum-thalamus-ventral striatum and inferior parietal lobule and correlated negatively in clusters including posterior insula—mediodorsal thalamus and midbrain. *Conclusion*. This study demonstrates that the temporal stability of perfusion MRI allows within-subject investigations of spontaneous fluctuations in mental state, such as mood, over relatively long-time intervals.

## 1. Background

Even though Parkinson's disease (PD) is a neurodegenerative disease defined by motor features [1], psychiatric sequelae are common such as depression, anxiety, and apathy [2, 3]. Previous studies have shown alteration of emotional processing in PD including reduced emotional physiologic response [4], impaired emotional word recognition [5], and impaired arousal judgment but normal valence [6]. The bulk of the evidence suggests that these changes result primarily from the degenerative process in the brain, and are not merely psychological reactions to disability [3]. Pathologically, Braak and Del Tredici [7] found that in PD clinical stages 1–3 (stage 4-5 pathologically), neurodegeneration could be seen in almost all areas of the brain including prefrontal cortex (PFC) and limbic system. Brain areas affected by PD that are hypothesized to cause emotional dysfunction including raphe nuclei, locus ceruleus, amygdala, mesolimbic, mesocortical, mesothalamic dopaminergic systems, and cingulate cortex [8]. Furthermore, neuroimaging studies have shown that a decrease in dopamine transporter availability in left putamen was associated with a reduction of ventrolateral prefrontal cortex activity during emotional gesture recognition tasks [9]. Lack of amygdala activation was observed by visual event-related potentials (ERPs) during facial expression recognition [4]. Based on these data, emotional processing in PD may differ from that of healthy controls. 

Most fMRI experiments on emotional processing used a variety of validated affective stimuli to elicit changes in mood; stimuli included pictures [10], sounds [11], and words [12]. These studies have identified various brain regions involved in the emotional responses to these stimuli, depending partly upon the type of stimulus [13, 14]. However, studies designed in this manner may identify brain regions involved in affective perception or naming rather than those that produce internal emotional states. Alternatively, the emotional states transiently induced by these artificial stimuli may be pale shadows of the emotional states people experience in response to spontaneous thoughts, real-life events, idiopathic mood disorders, or the cellular and pharmacological pathology of PD. 

We studied self-rated emotional valence and arousal in patients with PD on several occasions per subject, without attempting to induce specific emotional states. These patients were participating in a pharmacological perfusion MRI study of an adenosine A2a receptor antagonist and the dopamine precursor levodopa, but emotional ratings were obtained in the same drug and placebo conditions in each subject, allowing us to separate the effects of drug from spontaneous variance in emotional state across participants. The objective was to describe brain areas associated with naturalistic emotional state in PD. We hypothesized that spontaneous variation in self-rated emotional state would be accompanied by statistically significant changes in brain activity, as indexed by regional cerebral blood flow (rCBF) measured with perfusion MRI.

## 2. Materials and Methods

These data were collected during the course of a Phase IIa clinical and brain imaging study of the investigational adenosine A2a receptor antagonist SYN115, and the primary analyses of those data are reported elsewhere [15, 16]. The results presented here have not been previously reported except in abstract form [17].

### 2.1. Regulatory Approvals, Registrations, and Patient Consents

This study was approved by the Washington University Human Research Protection Office. Written documentation of informed consent was obtained in advance from each subject. Levodopa and SYN115 were given under US FDA Investigational New Drug application (IND) number 78,230.

### 2.2. Study Participants

Further details appear in Black et al. [15]. Briefly, 21 patients with Parkinson's disease (Hoehn and Yahr stages 1–3) on a stable dose of levodopa for 30 days were studied. Exclusion criteria included cognitive impairment indicated either by MMSE score <23 or estimated premorbid IQ <70 [18, 19], neurological diseases other than PD, self-reported history of psychosis or mania, current depression indicated by Geriatric Depression Scale Short Form [20] score >7, or current use of a dopamine agonist. All were Caucasian and right-handed, and 13 were male. Mean age was 60.8 years (range 44–73 years), mean duration of PD symptoms was 5.3 years (range 0.9–10.8 years), mean “off” UPDRS (placebo day, before levodopa) was 22.5 (range 7–51), and half had ever experienced dopa-induced dyskinesias.

### 2.3. Study Protocol

Participants were randomly assigned either (a) to take SYN115 twice daily for a week, wait 1 week (washout period), then take a matching placebo twice daily for a week, or (b) the reverse order. For 14 participants, each dose of active drug contained 60 mg of SYN115, whereas 12 subsequent subjects (5 of whom had participated in the 60 mg placebo study) received 20 mg at each dose. Participants and staff were blind to assignment.

On the last day of each treatment week, participants abstained from food, caffeine, and antiparkinsonian medication overnight, but took the last dose of SYN115 or placebo at 6 am at home. At the imaging center, they took 200 mg carbidopa, and then underwent a set of clinical and MRI assessments. An intravenous levodopa infusion was then begun, dosed in such a way as to rapidly produce and then maintain a steady plasma concentration [21], with a target concentration of 600 ng/mL. At least 25 minutes after the levodopa infusion started, all MRI and clinical assessments were repeated while the levodopa infusion continued. 

Participants rated their emotional state in each of four conditions: (1) before and (2) during levodopa infusion (after carbidopa) while taking oral SYN115, and (3) before and (4) during levodopa infusion while taking placebo pills. In each of these 4 conditions, 8 perfusion MRI (CBF) scans were acquired. In 4 of the 8 scans in each condition, the participant fixated on a crosshair throughout the entire 2.73 minutes of each scan; half of these were white on black and half were black on white. In 2 scans, an 8 Hz reversing circular checkerboard pattern surrounded the fixation crosshair, and in 2 scans the participant performed a 2-back letter working memory task for the entire scan.

### 2.4. Visual Analog Scale (VAS) and Scoring of Emotional Valence and Arousal

The circumplex model of emotion [22] describes human emotional states in terms of two independent constructs called valence and arousal (also called valence and “activation,” a term avoided here due to potential confusion with the homonymous word as used in the neuroimaging literature). The original model suggests but does not specify a numerical coordinate system for valence and arousal scores. For this study they were computed as follows. Participants rated various antipodal pairs of emotional descriptors from the circumplex model using VAS [23] displayed on a computer. They were instructed to freely self-evaluate their current feelings by clicking on the scale for each pair of emotional descriptors. The VAS ratings were recorded on 100 mm scales with anchor terms chosen from the original item set for 4 categories, that is, (a) negative valence, neutral arousal versus positive valence, neutral arousal (sad-happy, grouchy-cheerful); (b) negative valence, high arousal versus positive valence, low arousal (nervous-calm, distressed-relaxed); (c) negative valence, low arousal versus positive valence, high arousal (sluggish-lively, dull-excited); (d) neutral valence, high arousal versus neutral valence, low arousal (intense-tranquil, aroused-passive). For each VAS, the left item anchor was scored as 0 and the right anchor as 100. Subjects were advised to use the full 100 mm VAS range for each item and to score how they felt “at this moment.” Valence and arousal scores were computed from the VAS-item scores by the following formulae:

1. valence score = [average (a, b, c)/50]−1; possible range −1 to +1;
2. arousal score = 1−[average (b, 100−c, d)/50]; possible range −1 to +1.

### 2.5. MRI Methods

All MRI data were acquired on the Siemens 3T Tim Trio with matrix head coil. ASL images were acquired with the commercial Siemens pulsed arterial spin labeling (pASL) sequence [24]; the center-to-center slice distance was 7.5 mm. Details of image acquisition and transformation to scaled CBF images in atlas space are given elsewhere [15].

### 2.6. Statistical Analysis

Only those voxels were analyzed, that were represented in every EPI image in every subject. Statistical analysis of the CBF data was done via a two-level, random effects model using SPM8 software (http://www.fil.ion.ucl.ac.uk/spm/). First, a voxelwise general linear model (GLM) was computed for each subject. This first-level GLM followed the method of Henson and Penny [25] by including factors coding for each of the 16 possible combinations of drug (SYN115 or placebo), levodopa (before versus during infusion), and task (the 4 behavioral conditions described in Study Protocol, above). The GLM also included 3 covariates, representing the pertinent valence and arousal scores and their interaction. This approach partitions the variance from each subject's CBF data at a given voxel into components representing valence, arousal, and their interaction, plus components representing the nuisance variables drug, levodopa, task, and all their interactions. The *β* (model coefficient) images from each subject for the valence and arousal covariates became the input data for the final, second-level analysis.

The second-level (across-subjects) analysis was a voxelwise general linear model (GLM) that tested whether, across subjects, the mean *β* value for valence was significantly greater than zero, after controlling for sex, age, and dose group (60 versus 20 mg SYN115 b.i.d. during the active drug week). A corresponding analysis tested whether mean *β* was significantly *less* than 0. The same analyses were done for arousal. Multiple-comparisons correction was performed at the cluster level with the false discovery rate (FDR) set at *P* = 0.05. Approximate anatomical locations were provided by the Talairach Daemon client (http://www.talairach.org) [26], with corrections by reference to the study-specific MRI template atlas image.

## 3. Results

### 3.1. Valence and Arousal, and Their Association with Subject Characteristics

**Figure 1:**
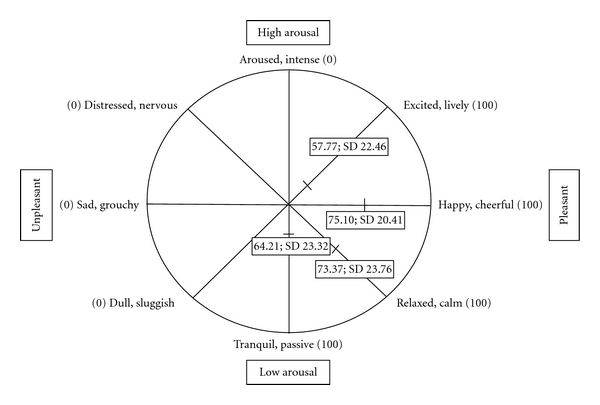
Score of visual analog scale on circumplex model of emotion. The 4 diameters shown represent the 8 pairs of adjectives used for the VAS items that generated the valence and arousal scores. The short perpendicular mark on each diameter represents the mean value for the corresponding VAS items in this sample.

The mean value for each VAS item and for the emotional valence and arousal scores are given in Table 1 and depicted on a diagram of the circumplex model of emotion (Figure 1). Across conditions, participants tended to have positive valence, mean 0.375 ± 0.339, and low arousal, mean −0.199 ± 0.254 (in other words, they tended to be closer to cheerful and calm than to the opposite). Valence and arousal scores correlated negatively with each other (*r* = −0.31, *P* < .01); that is, subjects who were less aroused (more tranquil) tended also to be happier. 

Table 2 shows associations of valence and arousal with pharmacological status and demographic variables. SYN115 increased valence (*t*(25) = 2.57, *P* = 0.02) and valence decreased from before to on levodopa (*t*(25) = −2.26, *P* = 0.03), though the mean change in valence score with either drug was less than 5% of the available range. Demographic variables and PD factors were not significantly associated with emotional valence or arousal (Table 2).

### 3.2. Perfusion MRI Data

#### 3.2.1. Correlations of rCBF with Valence

**Figure 2:**
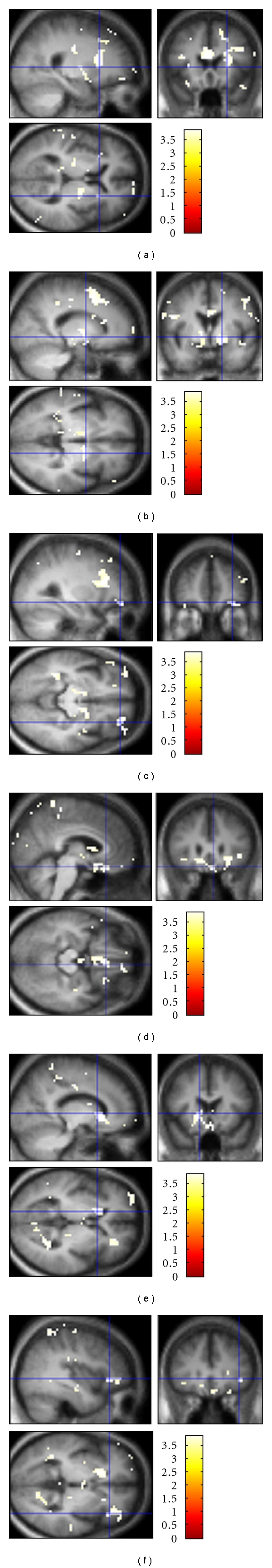
Statistical parametric (T) maps for correlations with valence. (a–c) Positive correlation with valence in (a) right putamen (22.5, 15, 6); (b) right medial globus pallidus (16.5, −3, −3); (c) right middle frontal gyrus (28.5, 42, −9) (BA 11). (d–f) Negative correlation with valence in (d) right subcallosal gyrus (4.5, 24, −15) (BA25); (e) left caudate (−13.5, 12, 3); (f) right inferior frontal gyrus (38, 27, 0) (BA47).

Random-effects analysis revealed significant positive correlations of rCBF with valence across subjects. We found significant areas in prefrontal-subcortical circuits; that is, bilateral dorsolateral PFC, bilateral anterior cingulate cortices (ACCs), orbital frontal cortex, striatum, and thalamus. Other significant clusters were observed in cortical areas including left and right ventral frontotemporal regions, lateral parietal cortex, insula, right motor, and premotor areas (see Table 3(a) and Figures 2(a)–2(c).

Areas whose rCBF correlated negatively with valence included a part of ACC, bilateral subcallosal cingulate cortex (SCC), along with parts of caudate and putamen, bilateral inferior frontal gyri, bilateral superior parietal lobule (SPL), inferior parietal lobule, precuneus, and PCC (see Table 3(b) and Figures 2(d)–2(f)).

#### 3.2.2. Correlations of rCBF with Arousal

**Figure 3:**
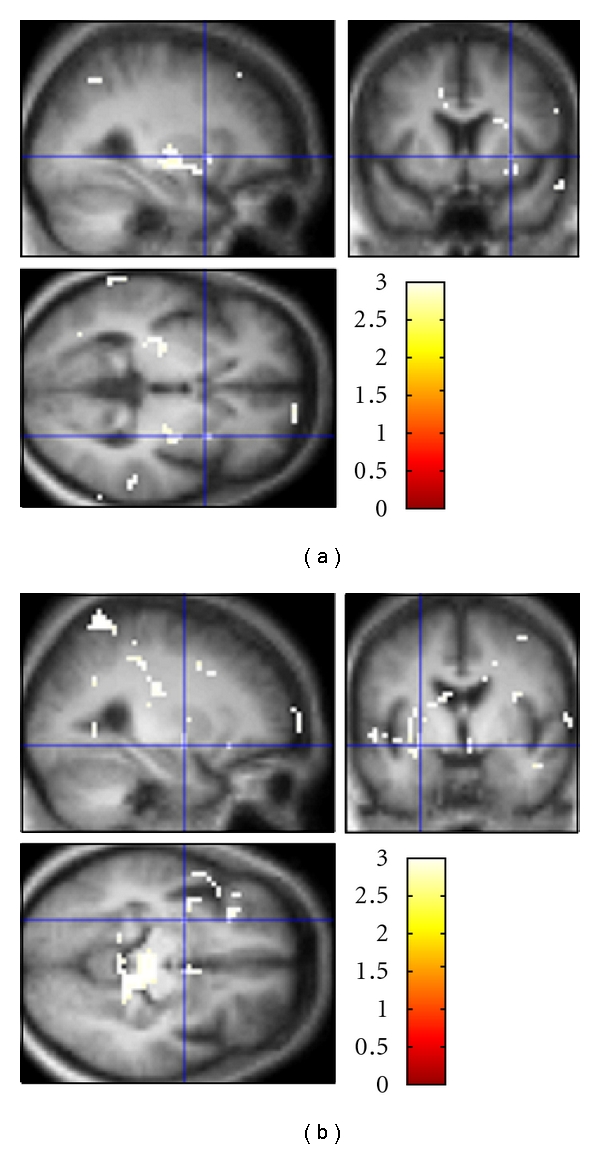
Statistical parametric (T) maps for correlations with arousal. (a) Positive correlation with arousal in right putamen (28.5, 6, 0). (b) Negative correlation with arousal in left amygdala-striatal transition area (−25.5, −6, −9).

Random-effects analysis of rCBF-arousal correlations found no voxels whose *t* value exceeded our predetermined voxel-level threshold corresponding to uncorrected *P* = 0.001. However, as an exploratory analysis, relaxing that initial threshold to a value corresponding to uncorrected *P* = 0.005 revealed several clusters that were significant after correction for multiple comparisons (Table 4 and Figures 3(a)-3(b)).

#### 3.2.3. Correlations of rCBF with the Interaction of Valence and Arousal

A random-effects analysis of the valence × arousal interaction found no activated clusters significant at *P* < 0.05 after correction for multiple comparisons.

## 4. Discussion

This study found a number of brain regions whose activity increased or decreased with changes in self-rated current mood state. Emotional state ratings, drew on the face validity and experimental history of the circumplex model of emotion [22], augmented here by a numerical implementation of the valence and arousal constructs. The conservative statistical approach employed for this analysis lends credence to the results and uses general linear modeling to minimize the potential confounds of demographic variation (age and sex) and unrelated experimental manipulations (such as medication status). Additionally, the study design allowed us to study ecologically valid or “real,” that is, spontaneously experienced, internal emotional state, which may more faithfully reflect patients' day-to-day experiences.

On the other hand, the study has a number of limitations, most of which derive from the fact that correlation of rCBF with current emotional state was not a primary goal of the data collection. Mood ratings were not done within the scanning session itself, but rather within a half hour or so, under broadly similar physiological conditions. This may have added noise to our results, so that we may have failed to detect some true correlations. Second, valence and arousal scores were (inversely) correlated, interpretation of results related to one emotional dimension might also be a result of changes in the other. However, the inclusion in our SPM model of a valence-arousal interaction should help disentangle their relation to rCBF. The correlation also tends to restrict the range of emotional states sampled. For that and perhaps other reasons, the range of emotional states reported by the subjects in our data did not equally sample all quadrants of emotional experience. Specifically, there was a bias of positive over negative valence, and low over high arousal. As a consequence, negative correlations with valence came primarily from data with positive values. Additionally, we cannot comment conclusively on whether regions identified as correlating with self-rated mood in this study are specific to PD, since we did not include healthy control subjects.

### 4.1. Nonimaging Results

Emotional rating in our participants tended towards positive valence and low arousal (Table 1, Figure 1). We did not test whether this differed from a control group. However, Drago et al. [6] show that non-demented PD patients under-rate arousal in others' facial expressions, compared to healthy control subjects, and that their spatial judgments are less affected than controls' by emotional stimuli. Imaging studies have shown decreased activation of emotional regions to emotional faces or gestures [9]. These findings may correspond to observations that PD patients actually experience lower emotional arousal, along with other manifestations of apathy [3, 4]. 

The slight improvement in mood with SYN115 is not surprising given that it is an adenosine 2a antagonist (caffeine is a nonspecific adenosine antagonist). The small decrease in valence on levodopa may seem counterintuitive, depression and anxiety commonly attend wearing off of individual levodopa doses in PD [27]. However, on-levodopa data were always collected a few hours after the off-levodopa data, and if subjects were merely less enthusiastic later in the study day, as one might expect, then valence and arousal would be lower, causing an apparent association with levodopa.

The lack of correlation of UPDRS with mood ratings may be a Type II error, but is consistent with other data suggesting that, contrary to common expectation, motor impairment is at best a modest predictor of mood state in PD [3].

### 4.2. Regions Associated with General Emotional Processing

Our study revealed a number of neural substrates associated with naturalistic emotional state; that is, (a) medial frontal PFC/ACC—subcortical circuit—medial PFC/ACC, basal ganglia, and thalamus; (b) limbic and paralimbic—amygdala, hippocampus and parahippocampal gyrus, thalamus, mamillary body, and PCC, insula, parietal, and lateral PFC; (c) visual system—occipital and temporal cortex. These regions are generally in line with previous studies in healthy controls. 

In a meta-analysis of functional neuroimaging studies of human emotions by Phan et al. [28, 29], medial frontal PFC (BA 9, 10) was activated in response to nonspecific emotion. In other words, this region was involved in emotional processing regardless of valence, arousal, or induction method. In the present study, we found some brain regions that contained activations associated with either arousal or valence, such as basal ganglia (BG), thalamus, and parietal lobe. Basal ganglia were correlated with happiness induction in 70% of the studies, and disgust induction in 60% [28] as well as responded to arousal stimuli evidenced by fMRI and skin conductance response (SCR) [30]. The thalamus is connected to BG, ACC, medial frontal PFC, orbital PFC, and dorsolateral PFC, forming several frontal-subcortical circuits [31]. The ACC is also closely interconnected to medial PFC. Lesions in the anterior cingulate—subcortical circuit can produce apathy [31], and the apathy experienced by PD patients who undergo subthalamic (STN)—deep brain stimulation (DBS) has been attributed to dysfunction of medial PFC [32, 33]. In addition, the thalamus links other structures in the limbic system, which is responsible for fundamental instinctive behaviors, cognition, and emotion, by receiving input from amygdala, basal forebrain, cerebellum, hippocampus, and septal nuclei, and projecting to prefrontal, cingulate, and parietal cortex [34]. Therefore, the BG, thalamus, and parietal cortex might be related to general emotion processing as a part of this network.

### 4.3. Associations between Valence and rCBF

We found regions in limbic and paralimbic structures—amygdala, medial PFC/rostral ACC, lateral PFC, and insula—that were positively correlated with valence; whereas subcallosal and posterior cingulate cortex (SCC and PCC) were negatively correlated with valence.

#### 4.3.1. Positive Correlation with Valence

Amygdala responses to valence or arousal stimuli have varied. Although amygdala response was related to arousal stimuli [35], and over 60% of studies reported amygdala activation in response to fear induction [28, 29], other studies have found activation to happy faces [36] or have linked amygdala activity to both valence and arousal [37, 38]. Thus, it may respond to salient characteristics of emotion. However, we found amygdala rCBF positively related only to valence. According to the neuropathological staging of PD (stages 1–6) proposed by Braak and colleagues [39], amygdala dysfunction first appears in presymptomatic stage 3 in a particular region. In addition, dopamine is lost in the amygdala due to degeneration of the ventral tegmental area in PD. In fact, loss of dopaminergic innervation of amygdala and other limbic structures were observed in PD subjects diagnosed with major depression [40], and dopamine modulates the response of amygdala to fearful stimuli in PD patients with depression [41]. 

The ACC is known to be involved in a form of attention that serves to regulate both cognitive (dorsal) and emotional (ventral) processings [42]. Valence perception has been reported to be normal in nondemented and nondepressed PD patients [6], thus, the activity in medial frontal PFC might reflect activation of circuits involved in valence-related attention or decision making.

#### 4.3.2. Negative Correlation with Valence

Subcallosal cingulate cortex was associated with sadness in about 46% studies [29], in line with our results. Clinically, patients with more than 3 episodes of untreated MDD had smaller SCC volume than controls [43, 44], and DBS of SCC may benefit treatment resistant depression [45].

Posterior cingulate cortex was also negatively correlated with valence. PCC has been linked to emotional processing and is thought to enhance memory for emotional stimuli [46]. A study that controlled for nonemotional, memory enhancing stimulus features suggested that this region might mediate interactions of emotional and memory-related processes [47]. Activity in PCC has also been reported to correlate with severity of anxiety symptoms in major depression and obsessive-compulsive disorder [48, 49] and was associated with levodopa dose-related mood fluctuations in PD patients [50].

### 4.4. Associations between Arousal and rCBF

We adopted a more permissive first-stage threshold to find any regions in which rCBF correlated with emotional arousal. The approach is reasonable, but as this threshold differs from the prespecified methods, the arousal results should be taken with a grain of salt.

The rCBF in hippocampus and middle temporal gyrus correlated positively with arousal in the present study, consistent with the study of Nielen et al. [51]. The connection with hippocampus may relate to observations that arousal can modulate memory [52]. 

Results from prior studies were arguable whether occipital lobes actually responded to valence or to arousal. The studies of Mourão-Miranda et al. [53] and Lane et al. [54] found that visual processing could vary with either valence or arousal, consistent with our findings, in which lingual gyrus was also associated negatively with valence, whereas others found occipital activation only when participants were presented stimuli of negative valence [51, 55]. The activation of the visual system with emotional valence and arousal may be that both can increase attentional processing. Emotional and attentional processings both involve medial frontal PFC, and a variety of functional neuroimaging studies have suggested that attention modulates activity of extra-striate visual cortex [55]. Moreover, threat stimuli lead to increased perceptual processing [56]. Our data might extend prior knowledge that current internal emotion, and not just visually presented emotional stimuli, may enhance visual system activity.

## 5. Conclusion

Emotions are usually regarded as brief but intense responses to changes in the environment featuring a number of subcomponents: (a) cognitive appraisal, (b) subjective feeling, (c) physiological response, (d) expression, (e) action tendency, and (f) regulation [57]. In addition, emotional stimulation can be of an interoceptive or exteroceptive nature. Different methods used to provoke emotional state changes can activate different systems. For example, the recall method activated mostly ACC and insula, whereas amygdala and occipital lobe were activated by visual induction [28].

Many neuroimaging studies of emotion in healthy volunteers have used clever methods to transiently stimulate emotional perception or attempt to quickly induce a given emotional state. Some experimental designs were chosen in part due to the limitations of blood oxygen level dependent (BOLD) fMRI, namely, its nonquantitative nature and the marked decline in signal-to-noise ratio of BOLD signal at time intervals greater than a few minutes.

The use of ASL perfusion fMRI enabled us to study within-subject fluctuations of internal mood states over relatively long periods of time (hours to weeks), a study design that is not possible with BOLD fMRI. Since we studied self-perceived emotional state without intentional provocation of a specific emotion, we could examine the effect of “subjective feeling” while being minimally confounded by other emotional processes. Furthermore, the rated emotion might be as a result of both “interoceptive” and “exteroceptive” natural stimulation that might result in stronger and more regions emotional stimulation, as compared to only one induction method alone. Despite the limitations of this study, it may demonstrate the potential utility of perfusion fMRI in the study of emotion.

## Figures and Tables

**Table 1:** Visual analog scale scores for each emotion item pair.

Visual analog scale	Mean, mm (range)
(1) Sad-happy	72.721 (8–100)
(2) Grouch-cheerful	77.471 (19–100)
(3) Nervous-calm	72.000 (5–100)
(4) Distressed-relaxed	74.731 (11–100)
(5) Sluggish-lively	55.010 (7–100)
(6) Dull-excited	60.529 (23–96)
(7) Intense-tranquil	67.375 (0–100)
(8) Aroused-passive	61.038 (1–100)
*Valence score *	0.375 (−0.333 to 0.967), SD 0.339
*Arousal score *	−0.199 (−0.673 to 0.423), SD 0.254

**Table 2:** Associations of valence and arousal with pharmacological status and demographic variables.

	Valence	Arousal	*P* (valence)	*P* (arousal)	*N* ^†^
Effect of SYN115 (mean difference [unitless])	+0.08	+0.05	0.02*	0.08	26
On levodopa minus pre-levodopa (mean difference [unitless])	−0.06	−0.04	0.03*	0.18	26
Age (correlation, *r*)	0.09	0.18	0.70	0.43	21
Hoehn and Yahr stage (correlation, *r*)	0.22	−0.03	0.34	0.89	21
UPDRS (correlation, *r*)	0.08	0.08	0.42	0.41	104
Sex (*t*-test)			0.82	0.92	
Male (mean)	0.33	−0.22			13
Female (mean)	0.30	−0.20			8
PD symptoms worse on which side of body? (*t*-test)			0.25	0.34	
Right (mean)	0.26	−0.17			13
Left (mean)	0.43	−0.28			7^‡^

*P ≤ 0.05.

^†^21 people, 26 experiments (5 people participated twice, once for the 20 mg b.i.d. study, once for the 60 mg b.i.d. study), 4 UPDRS measurements per experiment (26 × 4 = 104).

^‡^One subject was equally affected on left and right and was excluded from this analysis.

**Table:** (a) Clusters in the brain in which CBF correlates positively with valence

FDR-corrected *P* value	Number of voxels^a^	Peak *T* value (22 d.f.)	Coordinates of peak *T* value	Side of brain	Anatomical description of cluster
<10^−15^	674	3.86	28.5, 21, 18	Right	Middle frontal gyrus (BA 8, 9), precentral gyrus, anterior cingulate (BA 32), insula, putamen, caudate
0.001	76	3.86	4.5, 57, 18	Bilateral	Medial and superior frontal gyri (BA 10, 9)
0.001	97	3.86	−58.5, 3, 21	Left	Precentral gyrus (BA 6)
0.002	87	3.86	16.5, −3, −3	Right	Medial globus pallidus, ventrolateral thalamus, amygdala, frontal prepiriform cortex ventral to accumbens
0.002	82	3.86	−16.5, 6, −18	Left	Olfactory area, parahippocampal gyrus, medial globus pallidus/thalamus border, thalamus, hypothalamus, substantia nigra
0.053	36	3.85	28.5, 42, −9	Right	Middle frontal gyrus (BA 11)
0.010	56	3.84	−46.5, −60, 33	Left	Angular gyrus and inferior parietal lobule (BA 39, 40)

^a^Each voxel contained 0.027 mL.

**Table:** (b) Clusters in the brain in which CBF correlates negatively with valence

FDR-corrected *P* value	Number of voxels	Peak *T* value (22 d.f.)	Coordinates of peak *T* value	Side of brain	Anatomical description of cluster
<0.0002	117	3.86	−34.5, −42, 66	Left	Superior parietal lobule and precuneus (BA 7), postcentral gyrus (BA 5), posterior cingulate cortex (BA 31)
<10^−5^	178	3.86	−13.5, 12, 3	Bilateral	Subcallosal gyrus (BA 25), left caudate and putamen
0.007	67	3.86	4.5, −75, 54	Right	Superior parietal lobule and precuneus (BA 7), postcentral gyrus (BA 5)
0.020	46	3.86	−34.5, −15, 57	Left	Precentral gyrus (BA 6), postcentral gyrus (BA 3)
0.014	54	3.86	43.5, −39, 63	Right	Inferior parietal lobule (BA 40), superior parietal lobule (BA 7), postcentral gyrus (BA 5)
0.008	63	3.85	43.5, 39, −6	Right	Inferior frontal gyrus (BA 47)—lateral frontal part
0.020	46	3.85	25.5, −63, 6	Right	Lingual gyrus (BA 18, 19)
0.036	39	3.85	−37.5, 33, −6	Left	Inferior frontal gyrus (BA 47)
0.043	36	3.83	25.5, 24, −6	Right	Inferior frontal gyrus (BA 47)

Note: Only clusters significant after correction for multiple comparisons are shown here.

**Table:** (a) Clusters in the brain in which CBF correlates positively with emotional arousal^†^

FDR-corrected *P* value	Number of voxels	Peak *T* value (22 d.f.)	Coordinates of peak *T* value	Side of brain	Anatomical description of cluster
0.039	84	3.00	31.5, −9, −6	Right	Putamen, thalamus (ventrolateral), hippocampus
0.039	85	3.00	−34.5, −75, 6	Left	Middle temporal gyrus (BA 39), middle occipital gyrus (BA 19)
<0.0005	231	3.00	−37.5, −57, 45	Left	Inferior parietal lobule (BA 40), superior occipital gyrus (BA 19)
0.039	100	3.00	34.5, −57, 39	Right	Inferior parietal lobule (BA 40), superior parietal lobule and precuneus (BA 7)

**Table:** (b) Clusters in the brain in which CBF correlates negatively with emotional arousal^†^

FDR-corrected *P* value	Number of voxels	Peak *T* value (22 d.f.)	Coordinates of peak *T* value	Side of brain	Anatomical description of cluster
0.001	216	3.00	−34.5, −27, 27	Left	Striatum and nearby white matter, thalamus (medial dorsal), insula (BA 13)
0.015	110	3.00	−34.5, −60, 57	Left	Precuneus and superior parietal lobule (BA 7)
0.005	139	3.00	−25.5, −6, −9	Left	Putamen, claustrum, insula (BA 13), amygdala-striatal transition area, superior temporal gyrus (BA 22, 38), inferior frontal gyrus (BA 47)—ventral frontal part
<10^−7^	577	3.00	13.5, −45, −6	Right	Superior and middle occipital gyrus, cuneus (BA 17, 18, 19), posterior cingulate cortex (BA 23), thalamus (medial dorsal), midbrain (central portion and red nucleus)
0.005	149	3.00	34.5, −81, 39	Right	Superior parietal lobule and precuneus (BA 7)

^†^No voxels passed the predefined voxel-level threshold of *P* < 0.001. For hypothesis generation, we repeated our analysis using a voxel-level threshold of *P* < 0.005, and those results are shown here.

Note: only clusters significant after correction for multiple comparisons are shown here.
